# The associations of the triglyceride-glucose index and its combination with blood pressure on cardiovascular and all-cause mortality in hypertension: a national study

**DOI:** 10.3389/fendo.2024.1469055

**Published:** 2024-10-14

**Authors:** Jun-Peng Xu, Xiong-Qiang Peng, Li-Heng Guo, Xu-Jie Zhao, Mao-Sheng Chen, Xiao-Yi Mai, Jia-Wei Tan, Quanfu Chen, Rui-Xiang Zeng, Min-Zhou Zhang

**Affiliations:** ^1^ The Second Clinical College of Guangzhou University of Chinese Medicine, Guangzhou, China; ^2^ Department of Critical Care Medicine, Guangdong Provincial Hospital of Chinese Medicine, Guangzhou, China

**Keywords:** triglyceride-glucose index, insulin resistance, blood pressure, mortality, joint analyses

## Abstract

The triglyceride-glucose (TyG) index, a reliable surrogate biomarker of insulin resistance (IR), is highlighted recently as related to cardiac disorders. However, the associations of the TyG index and its combination with blood pressure (BP) on cardiovascular and all-cause mortality in hypertension remain unclear. In this study, we included 9,635 hypertension patients from the National Health and Nutrition Examination Survey 1999–2018. During a median follow-up of 8.00 years, a total number of 853 and 2,489 in cardiovascular and all-cause deaths occurred, respectively. After full adjustments, the individual TyG index was positively associated with cardiovascular and all-cause mortality with J-shaped dose–response relationships. The lowest risk thresholds of the TyG index for cardiovascular and all-cause mortality were 9.5551 and 9.3405, respectively. In joint analyses, the highest risks of cardiovascular and all-cause mortality were observed among those with elevated levels of both BP and IR [1.64 (1.18, 2.28), *P* = 0.0031; 1.39 (1.14, 1.70), *P* = 0.0011; respectively]. In sensitivity and subgroup analyses, the results were generally robust. Our data appeal a hypothesis that future treatments for hypertension may require a combination of BP controlled and IR improvement.

## Introduction

Hypertension is a major factor that contributes to cardiovascular disease (CVD), comorbidities, and mortality worldwide (1). Recent data have revealed that there are approximately 1.13 billion people living with hypertension and that the total costs attributed to hypertension from 2010 to 2030 may reach US$400 billion; thus, hypertension causes enormous global disease and economic burdens (2, 3). Although hypertension awareness and hypertension treatments have improved over time, substantial work is still needed. For example, the blood pressure (BP) of more than half of patients with either treated or untreated hypertension is not under control (4). On the one hand, there is a consensus that uncontrolled hypertension, regardless of the use of antihypertensive therapy, is closely related to increased risk of CVD (5). Specifically, every elevation of 20 mmHg or 10 mmHg in systolic or diastolic BP is associated with an approximately twofold increased risk of CVD (6). On the other hand, some risk factors, such as obesity and insulin resistance (IR), can trigger excessive activation of the renin–angiotensin–aldosterone system (RAAS) or the sympathetic nervous system, resulting in worse BP and hypertension progression (7). Furthermore, hypertension in individuals with normal BP should not be mistaken as a low-risk factor; otherwise, residual risks of adverse outcomes may be overlooked. Collectively, identifying and evaluating individuals with uncontrolled BP and concomitant residual risk factors is imperative for preventing disease and reducing mortality.

A previous study indicated that insulin resistance (IR) was directly correlated with elevated systolic and diastolic BP, particularly in untreated patients with essential hypertension (8). Additionally, patients who had hypertension at the time of diabetes diagnosis had increased risks of CVD and all-cause death than diabetic patients with normotension (9). These findings suggest that numerous intrinsic associations are shared between IR and hypertension. Recently, a reliable surrogate biomarker of IR, the triglyceride−glucose (TyG) index (which is calculated from fasting blood glucose and triglyceride levels), has been shown to be related to cardiac and noncardiac disorders (4, 10). Evidence from epidemiological data has shown that, in the general population, the combination of a low TyG index and low systolic BP (<120 mmHg and <130 mmHg) was associated with reduced risks of CVD and all-cause mortality, which was not observed in a group with both a low TyG index and SBP < 140 mmHg (11). However, whether the results are appropriate for patients with hypertension remains unknown. Furthermore, this study did not add diastolic BP to the combined investigation of the TyG index and BP, which might have resulted in incomplete practicality of the conclusions, as diastolic BP also has a great impact on mortality (12). Moreover, a lower number of events (61 CVD and 238 all-cause deaths) among the 6,245 subjects in that study might have led to potential statistical bias. Thus, we conducted a large-scale epidemiological analysis from the National Health and Nutrition Examination Survey (NHANES) to better understand the associations of the combination of the TyG index and BP on cardiovascular and all-cause mortality in hypertension patients, with the aim of providing more evidence to both identify potential biomarkers and further improve risk stratification of the disease.

## Materials and methods

### Study population

Publicly available data from the NHANES, a nationally representative sample of non-institutionalized US civilian populations, were used in the present study. The detailed design and procedure of NHANES are described on the website (https://www.cdc.gov/nchs/nhanes/). The protocols of the NHANES study were approved by the Institutional Review Board of the National Center of Health Statistics, and informed written consent was provided by each participant upon enrolment. The sampling methods and analytic guidelines of the NHANES have been previously published (13). This study adhered to the reporting guidelines for cohort studies, the Strengthening the Reporting of Observational Studies in Epidemiology (STROBE).

The NHANES data were collected from 1999 to 2018. According to the American Heart Association (AHA) new guidelines of 2018 (14), we initially defined hypertension on the basis of the following criteria (1): a self-reported physician-diagnosed history of hypertension (2); receipt of oral antihypertensive therapy, including calcium channel blockers, angiotensin-converting enzyme inhibitors, angiotensin receptor blockers, β-receptor antagonists, and diuretics; and (3) record of 3 non-same-day randomized systolic blood pressure (SBP) values ≥130 mmHg or diastolic blood pressure (DBP) values ≥80 mmHg at baseline. However, in subsequent major analyses, we still used the common hypertension diagnosis criteria, including the former two criteria and the traditional BP value, i.e., a hypertensive cut-off value of 140/90 mmHg. As described by the protocol, all blood pressure measurements were obtained at a mobile examination centre, and each blood pressure was calculated as a mean value of three or four consecutive right-hand readings by a mercury sphygmomanometer after the patient had rested quietly in a seated position for 5 min. Initially, the total number of participants from NHANES 1999–2018 was 101,316. We excluded subjects who were aged ≤20 years (n = 47,208), pregnant (n = 1,469), whose hypertension status was unavailable on the basis of the AHA new criteria (n = 24, 143), whose blood pressure (n = 1,866) and TyG index (n = 14,735) were unavailable, or who were lost to follow-up (n = 14); 11,881 participants were included in the sensitivity analysis. Finally, after excluding those with hypertension according to the hypertensive cut-off value of 130/80 mmHg, 9,635 subjects were enrolled (**Supplementary Figure S1**).

### Covariate assessment

Covariates such as age, sex, ethnicity, education, family income-to-poverty ratio, current smoking status, current alcohol consumption, physical activity, disease conditions, and related therapeutic drug administration were obtained from standardized questionnaires during the household interviews. Information on body weight and height was collected at a mobile examination centre. The body mass index was calculated as weight in kilograms divided by height in metres squared (kg/m^2^) and was categorized as lower than 25, 25 to 30, or greater than 30 (15). A family income-to-poverty ratio <1.00 indicated that the household income was below the poverty threshold, and a ratio >3.00 suggested that the household income was more than triple the poverty threshold. Participants were classified as inactive (does not meet guidelines, <7.5 metabolic equivalents of task) or active (meets guidelines, ≥7.5 metabolic equivalents of task) on the basis of the self-reported volume of moderate or vigorous recreational physical activity per week (16, 17). In addition, current smokers were identified on the basis of self-reported answers such as “Smoked at least 100 cigarettes in life” and “Do you currently smoke cigarettes?”. Current alcohol consumption was defined as the consumption of at least 12 alcohol drinks during the past year. In addition, in the present study, cardiovascular diseases were included and defined as any self-reported physician-diagnosed history of stroke, angina pectoris, myocardial infarction, coronary heart disease, or chronic heart failure (18). The estimated glomerular filtration rate (eGFR) was calculated to evaluate the kidney function of each participant following the Chronic Kidney Disease Epidemiology Collaboration equation (19), and we accordingly defined chronic kidney disease as an eGFR <60 ml/min/1.73 m^2^.

### Determination of mortality

The follow-up information of deaths was acquired from the National Death Index through 31/12/2019, on the website (https://www.cdc.gov/nchs/ndi/). According to the International Classification of Diseases, 10th Clinical Modification (ICD-10) system codes, we defined cardiovascular mortality with the codes I00–I09, I11, I13, I20–I51 and I60–I69. Death due to any cause was included in the definition of all-cause mortality.

### Statistical analysis

In the present study, all analyses incorporated sample weights, clustering, and stratification to ensure nationally representative estimates. The major analyses were performed in the following three steps. First, 9,635 hypertension participants were divided into three groups according to different baseline blood pressure values. Group 1 patients with well-controlled hypertension were defined as those whose SBP values were <140 mmHg and whose DBP values were <90 mmHg. Group 2 included patients whose SBP values ranged from 140 mmHg to <160 mmHg and whose DBP values were <100 mmHg or whose DBP values ranged from 90 mmHg to <100 mmHg and whose SBP values were <160 mmHg. Those in group 3 had SBP values ≥160 mmHg or DBP values ≥100 mmHg. In parallel, all participants were divided by tertile cut-off points of the baseline TyG index to minimize any bias. Data are presented as weighted means ± standard errors for continuous variables and as weighted frequencies or percentages for categorical variables. We used the Kruskal−Wallis test and the χ^2^ test for continuous and categorical variables, respectively, to estimate the differences among those groups. To explore the dose−response relationships between the baseline TyG index and mortality, restricted cubic spline curves with three knots (10th, 50th, and 90th) were generated. If the relationship was non-linear, we estimated the threshold values with multivariable Cox proportional hazards regression models and defined categorical groups of the TyG index, where appropriate Kaplan−Meier plots were generated to estimate the cumulative hazards, and log-rank tests were applied to examine the differences between the lower and higher TyG index level groups. We then used a two-piecewise Cox proportional risk model on both sides of the inflection point to investigate the associations of the TyG index with the risk of mortality and conducted log likelihood ratio tests to confirm the non-linearity. To further investigate the associations between the baseline TyG index and mortality, we constructed multivariable Cox proportional hazards regression models to calculate hazard ratios (HRs) and corresponding 95% confidence intervals (CIs). Schoenfeld residuals were performed to assess the proportional hazards assumption, and no violation was observed. Model I was adjusted for age (continuous, years), sex (female or male), ethnicity (Mexican American, non-Hispanic Black, non-Hispanic White, or other, which included other Hispanic), family income to poverty ratio (<1.0, 1.0–3.0, >3.0, or missing), education (lower than high school, high school or equivalent, or more than high school), and marital status (married, divorced/separated/widowed, unmarried/cohabitating). In model II, we further adjusted for body mass index (<25.0, 25.0–30.0, >30.0), current smoking status, current drinking status, diabetes status, cardiovascular diseases, hyperlipidaemia, chronic kidney disease, cancer, moderate-to-vigorous physical activity (inactive or active), hypoglycaemic agents (oral drugs and insulin injection), antithrombosis drugs (aspirin and clopidogrel), statins, and oral antihypertensive therapy. Additionally, we performed analyses adjusting for several metabolic factors, including blood pressure (continuous, mmHg), blood lipids (continuous, mg/dl), and HbA1c (continuous).

Second, Spearman correlation coefficients were used to address associations between BP and the TyG index. Next, we redistributed the participants into four groups for joint analyses of baseline blood pressure and the TyG index for mortality. According to previous residual risk studies (11, 20), we divided participants as follows: those with no residual risk, SBP <140 mmHg and DBP <90 mmHg, and TyG index < estimated threshold; residual blood pressure risk (RBR), SBP ≥140 mmHg or DBP ≥90 mmHg, and TyG index < estimated threshold; residual insulin resistance risk (RIRR), SBP <140 mmHg and DBP <90 mmHg, and TyG index ≥ estimated threshold; residual blood pressure and insulin resistance risk (RBIRR), SBP ≥140 mmHg or DBP ≥90 mmHg, and TyG index ≥ estimated threshold. Kaplan–Meier survival curves were constructed to visualize the relationships between each joint group and subsequent mortality risk over time. Similarly, three multivariable Cox proportional hazards regression models were performed.

Third, several sensitivity and subgroup analyses with fully adjusted multivariable Cox proportional hazards regression models were performed to evaluate the robustness of the findings. For example, we excluded participants who died within 1 year or whose follow-up time was less than 1 year and reanalysed. Another sensitivity analysis was performed by using a new hypertensive cut-off value of 130/80 mmHg according to the AHA guidelines (14). In addition, we performed subgroup analyses stratified by obesity, diabetes, and antihypertensive therapy.

A two-sided *P* < 0.05 was considered statistically significant. All the analyses were performed with the statistical software package R (http://www.R-project.org, The R Foundation).

## Results

### Baseline characteristics

**Tables 1**, **2** summarize the weighted demographic and clinical characteristics of the enrolled subjects by increasing tertiles of BP and the TyG index, respectively. The prevalence of women, normal BMI, poverty, non-Hispanic Black, divorced/separated/widowed, non-drinkers, and non-smokers was generally higher among those with increased BP, whereas the prevalence of men, overweight individuals, non-Hispanic White individuals, married individuals, individuals who consumed alcohol, individuals who smoked, and individuals taking medications was higher in those with elevated TyG indices. Additionally, those who were older and had a low level of education, inadequate moderate-to-vigorous physical activity, and a history of chronic disease had higher BP and TyG indices. During the 8.00-year median follow-up period, 853 and 2,489 cardiovascular and all-cause deaths occurred, respectively. The number of deaths in each tertile group is shown in **Tables 1**, **2**.

**Table 1:** Baseline characteristics of hypertension participants by blood pressure levels in NHANES from 1999 to 2018^a^.

	Participants, no. (%)			
	Blood pressure levels			
Characteristics	Tertile 1^b^	Tertile 2^c^	Tertile 3^d^	*P*-value
Participants, no.	5144	3163	1328	
SBP, mean (SE), mmHg	121.58 (0.20)	144.45 (0.23)	170.69 (0.55)	<0.001
DBP, mean (SE), mmHg	69.73 (0.24)	78.00 (0.36)	82.03 (0.77)	<0.001
Triglyceride-glucose index, mean (SE)	8.82 (0.01)	8.86 (0.02)	8.86 (0.02)	0.087
Fasting triglyceride, mean (SE), mg/dl	148.54 (2.54)	153.46 (2.44)	148.82 (3.63)	0.303
Total cholesterol, mean (SE), mg/dl	193.13 (0.93)	203.72 (0.97)	205.65 (1.94)	<0.001
Fasting blood glucose, mean (SE), mg/dl	113.56 (0.69)	114.39 (0.95)	116.19 (1.38)	0.263
Glycated haemoglobin (HbA1c), mean (SE)	5.87 (0.02)	5.83 (0.02)	5.97 (0.04)	0.309
Age, mean (SE), years	55.63 (0.29)	58.04 (0.37)	64.33 (0.51)	<0.001
Male	2,512 (48.78)	1,678 (53.09)	597 (44.07)	<0.001
Body mass index				<0.001
<25.0	874 (16.89)	719 (21.82)	335 (25.41)	
25.0 to 30.0	1,753 (33.99)	1,120 (33.70)	552 (36.72)	
>30.0	2,517 (49.12)	1,324 (44.48)	471 (37.87)	
Family income, income/poverty ratio				<0.001
<1.0	893 (11.41)	515 (11.73)	305 (17.82)	
1.0 to 3.0	2,007 (34.28)	1,372 (37.82)	554 (39.56)	
>3.0	1,805 (47.85)	991 (43.65)	312 (32.07)	
Missing	439 (6.46)	285 (6.80)	157 (10.55)	
Race/ethnicity				<0.001
Mexican American	635 (4.79)	499 (6.09)	240 (5.98)	
Non-Hispanic White	2,558 (74.01)	1,398 (69.69)	521 (62.39)	
Non-Hispanic Black	1,162 (10.94)	791 (13.80)	365 (17.45)	
Other	789 (10.26)	475 (10.42)	202 (13.19)	
Education				<0.001
Lower than high school	1,419 (18.50)	1,004 (20.43)	530 (28.65)	
High school	1,227 (25.02)	817 (28.94)	320 (28.08)	
More than high school	2,498 (56.48)	1,342 (50.63)	478 (43.27)	
Marital status				<0.001
Married	2,829 (59.97)	1,739 (58.70)	639 (50.69)	
Divorced/separated/widowed	1,488 (23.91)	905 (24.52)	494 (33.36)	
Unmarried/cohabitation	827 (16.12)	519 (16.78)	195 (15.95)	
Disease condition				
Diabetes mellitus	1,191 (18.81)	580 (14.30)	298 (18.22)	<0.001
Cardiovascular diseases	1,129 (18.59)	543 (14.74)	314 (22.46)	<0.001
Hyperlipidaemia	3,493 (68.97)	2,138 (67.69)	904 (68.29)	0.619
Chronic kidney disease	807 (12.61)	466 (12.77)	303 (22.27)	<0.001
Cancer	728 (14.86)	422 (12.41)	182 (16.52)	0.090
Current smoking	2,654 (52.46)	1,538 (49.81)	621 (46.45)	0.011
Current drinking	2,978 (62.47)	1,783 (59.30)	628 (48.98)	<0.001
Physical activity				<0.001
Does not meet guidelines	3,996 (75.22)	2,589 (79.19)	1,151 (85.60)	
Meets guidelines	1,148 (24.78)	574 (20.81)	177 (14.40)	
Hypoglycaemic agents	1,095 (17.27)	518 (12.88)	260 (15.85)	<0.001
Antithrombosis drugs	339 (5.31)	136 (3.60)	80 (5.00)	0.008
Statin	1,816 (33.58)	788 (24.06)	336 (27.48)	<0.001
Antihypertensive therapy	3,760 (70.97)	1,517 (44.66)	732 (52.62)	<0.001
Outcomes				
Cardiovascular mortality	355 (5.88)	285 (8.35)	213 (18.36)	<0.001
All-cause mortality	1,112 (17.15)	842 (21.89)	544 (36.47)	<0.001

SE, standard error; SBP, systolic blood pressure; DBP, diastolic blood pressure; NHANES, National Health and Nutrition Examination Survey. Cardiovascular diseases included myocardial infarction, congestive heart failure, coronary heart disease, angina pectoris, and stroke. Hypoglycaemic agents included oral drugs and insulin injection. Antithrombosis drugs included aspirin and clopidogrel. Antihypertensive therapy included angiotensin-converting enzyme inhibitors, angiotensin-II receptor blockers, calcium channel blockers, β-blockers and diuretics.

a All estimates accounted for complex survey designs, and all percentages were weighted.

b Participants with SBP <140 mmHg and DBP <90 mmHg.

c Participants with SBP from 140 mmHg to < 160 mmHg and DBP < 100 mmHg or DBP from 90 mmHg to <100 mmHg and SBP <160 mmHg.

d Participants with SBP >160 mmHg or DBP >100 mmHg.

**Table 2:** Baseline characteristics of hypertension participants by TyG index levels in NHANES from 1999 to 2018^a^.

	Participants, no. (%)			
	Triglyceride-glucose (TyG) index levels			
Characteristics	Tertile 1	Tertile 2	Tertile 3	*P*-value
Participants, no.	3212	3211	3212	
Triglyceride-glucose index, mean (SE)	8.14 (0.01)	8.78 (0.00)	9.56 (0.01)	<0.001
Fasting triglyceride, mean (SE), mg/dl	71.95 (0.51)	125.25 (0.57)	248.78 (3.71)	<0.001
Total cholesterol, mean (SE), mg/dl	186.09 (1.02)	198.11 (0.90)	208.42 (1.12)	<0.001
Fasting blood glucose, mean (SE), mg/dl	99.90 (0.34)	107.21 (0.47)	134.40 (1.32)	<0.001
Glycated haemoglobin (HbA1c), mean (SE)	5.53 (0.01)	5.70 (0.02)	6.36 (0.04)	<0.001
SBP, mean (SE), mmHg	133.72 (0.46)	133.07 (0.42)	134.99 (0.46)	0.046
DBP, mean (SE), mmHg	72.79 (0.33)	73.61 (0.38)	74.41 (0.34)	<0.001
Age, mean (SE), years	56.58 (0.38)	57.70 (0.37)	57.58 (0.31)	0.026
Male	1,529 (45.52)	1,556 (49.12)	1,702 (54.05)	<0.001
Body mass index				<0.001
<25.0	970 (31.34)	603 (17.58)	355 (9.80)	
25.0 to 30.0	1,114 (35.58)	1,164 (34.39)	1,117 (32.68)	
>30.0	1,128 (33.09)	1,444 (48.03)	1,740 (57.52)	
Family income, income/poverty ratio				0.199
<1.0	569 (13.00)	547 (11.76)	597 (11.84)	
1.0 to 3.0	1,261 (33.46)	1,313 (36.52)	1,358 (37.72)	
>3.0	1,079 (46.22)	1,068 (45.05)	961 (43.41)	
Missing	302 (7.32)	283 (6.67)	296 (7.03)	
Race/ethnicity				<0.001
Mexican American	294 (4.41)	478 (5.73)	602 (6.34)	
Non-Hispanic White	1,291 (65.41)	1,553 (73.07)	1,633 (75.44)	
Non-Hispanic Black	1,205 (20.76)	651 (10.04)	462 (7.27)	
Other	422 (9.69)	529 (11.17)	515 (10.95)	
Education				<0.001
Lower than high school	860 (17.82)	998 (21.26)	1,095 (21.28)	
High school	790 (24.96)	752 (25.77)	822 (28.90)	
More than high school	1,562 (57.23)	1,461 (52.96)	1,295 (49.81)	
Marital status				0.010
Married	1,614 (55.68)	1,800 (59.78)	1,793 (60.15)	
Divorced/separated/widowed	979 (25.77)	950 (24.80)	958 (24.76)	
Unmarried/cohabitation	619 (18.55)	461 (15.42)	461 (15.09)	
Disease condition				
Diabetes mellitus	347 (7.78)	554 (12.93)	1,168 (30.75)	<0.001
Cardiovascular diseases	588 (15.20)	627 (16.71)	771 (21.31)	<0.001
Hyperlipidaemia	1,717 (52.24)	2,079 (66.00)	2,739 (86.33)	<0.001
Chronic kidney disease	460 (12.01)	540 (14.26)	576 (14.65)	0.027
Cancer	434 (14.20)	447 (14.94)	451 (14.56)	0.828
Current smoking	1,531 (48.29)	1,547 (49.64)	1,735 (54.91)	<0.001
Current drinking	1770 (60.01)	1790 (59.83)	1829 (60.33)	0.009
Physical activity				<0.001
Does not meet guidelines	2,437 (72.53)	2,574 (77.38)	2,725 (82.46)	
Meets guidelines	775 (27.47)	637 (22.62)	487 (17.54)	
Hypoglycaemic agents	307 (6.85)	492 (11.72)	1,074 (28.16)	<0.001
Antithrombosis drugs	159 (4.25)	179 (4.29)	217 (5.66)	0.072
Statin	879 (26.30)	956 (29.39)	1,105 (33.95)	<0.001
Antihypertensive therapy	1,859 (54.53)	1,989 (61.37)	2,161 (66.06)	<0.001
Outcomes				
Cardiovascular mortality	239 (6.23)	282 (7.58)	332 (9.66)	<0.001
All-cause mortality	729 (17.74)	820 (20.01)	949 (24.10)	<0.001

SE, standard error; SBP, systolic blood pressure; DBP, diastolic blood pressure; NHANES, National Health and Nutrition Examination Survey. Cardiovascular diseases included myocardial infarction, congestive heart failure, coronary heart disease, angina pectoris and stroke. Hypoglycaemic agents included oral drugs and insulin injection. Antithrombosis drugs included aspirin and clopidogrel. Antihypertensive therapy included angiotensin-converting enzyme inhibitors, angiotensin-II receptor blockers, calcium channel blockers, β-blockers and diuretics.

^a^All estimates accounted for complex survey designs, and all percentages were weighted.

The results of the Spearman correlation analyses are shown in the **Supplementary Table S1**, and indicate positive associations of the TyG index with BMI, triglyceride, blood glucose, total cholesterol, low-density lipoprotein cholesterol, glycated haemoglobin and blood pressure (all *P* < 0.05), whereas inverse correlations with high-density lipoprotein cholesterol, eGFR, and moderate-to-vigorous physical activity were detected (all *P* < 0.05).

### Associations of the TyG index with cardiovascular and all-cause mortality

**Figure 1:**
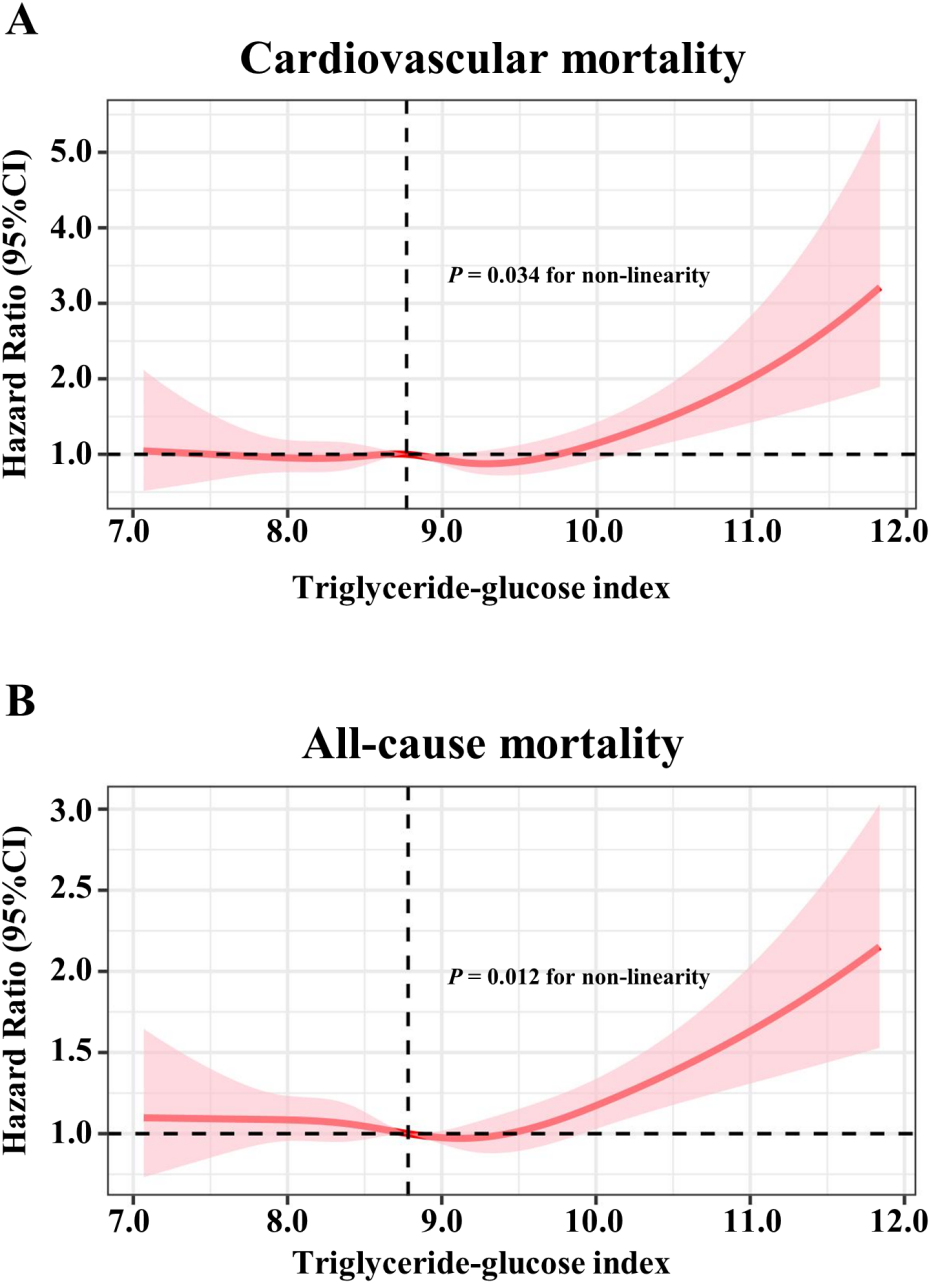
Restricted cubic spline models for the association between triglyceride-glucose index and mortality. Adjusted hazards ratio (solid pink lines) and 95% confidence intervals (pink shadow) after controlling covariates in **Table 1** for **(A)** cardiovascular mortality, **(B)** all-cause mortality. Splines were examined in the fully adjusted model with the best placed knots at the 10th, 50th, and 90th percentiles of triglyceride-glucose index.

**Figure 2:**
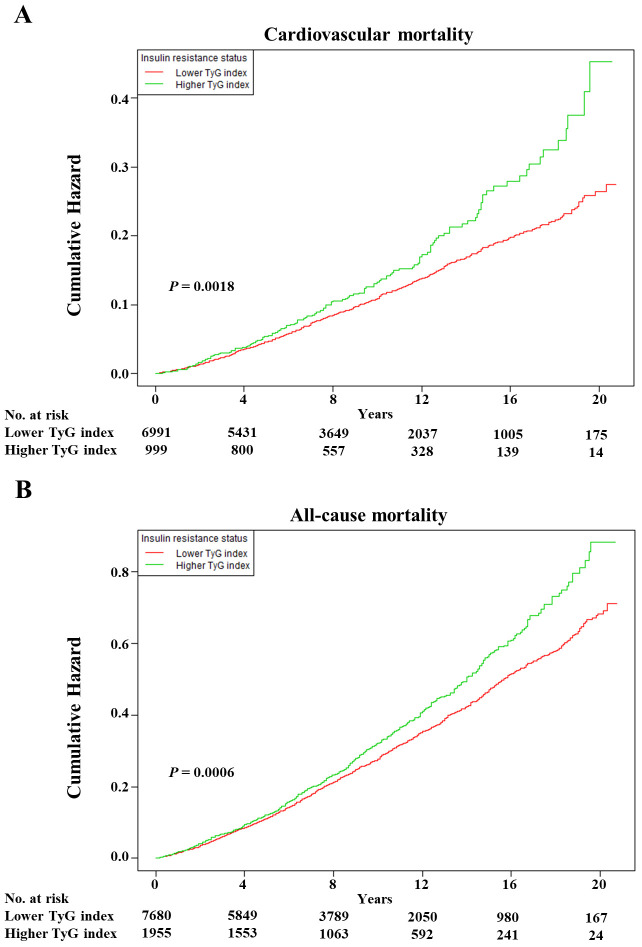
The Kaplan–Meier curves after controlling covariates in **Table 1** for cumulative hazards of the mortality in individual TyG index analysis. **(A)** Cardiovascular mortality, **(B)** all-cause mortality. TyG index, triglyceride-glucose index. Lower TyG index was defined by TyG index <9.5551 and <9.3405 in cardiovascular and all-cause mortality, respectively. Higher TyG index was defined by TyG index ≥9.5551 and ≥9.3405 in cardiovascular and all-cause mortality, respectively.

As shown in **Figure 1**, the multivariable restricted cubic spline curves revealed approximately J-shaped dose−response relationships between the TyG index and cardiovascular and all-cause mortality. Consistently, the results of two piecewise linear regression model tests confirmed these non-linear associations (both *P* values for log-likelihood ratio tests < 0.001, **Supplementary Table S2**). Notably, the threshold values of the TyG index related to the lowest risk were 9.5551 and 9.3405 for cardiovascular mortality and all-cause mortality, respectively. Kaplan−Meier survival curves were generated according to the threshold values. Elevated cumulative risks were observed in the higher TyG index group in terms of cardiovascular and all-cause mortality (*P* = 0.0018 and *P* = 0.0006, respectively; **Figure 2**). Similarly, when participants with lower TyG indices (below the threshold) were treated as a reference in the categorical analyses, the HRs and corresponding 95% CIs of cardiovascular and all-cause mortality in those with higher TyG indices were 1.56 (1.22, 1.99, *P* =0.0003) and 1.20 (1.05, 1.38, *P* =0.0086) after adjusting for age, sex, family income, BMI, race, education and marital status in model I (**Table 3**). In model II (additionally adjusted for smoking, alcohol consumption, chronic diseases, physical activity, and medications), the HRs and corresponding 95% CIs of cardiovascular and all-cause mortality in participants with higher TyG indices were still positive [1.43 (1.10, 1.85, *P* =0.0067) and 1.17 (1.02, 1.34, *P* =0.0257), respectively]. Furthermore, in continuous analyses, each 1-increment increase in the TyG index was positively associated with a 29% and 11% increased risk of cardiovascular and all-cause mortality, respectively. After additional adjustment for metabolic factors, including blood pressure, blood lipids, and HbA1c, the predictive effect of the TyG index on cardiovascular and all-cause mortality remained statistically significant [1.18 (1.00, 1.41, *P* =0.0488) and 1.11 (1.01, 1.22, *P* =0.0339), respectively].

**Table 3:** Hazard ratios of TyG index for cardiovascular and all-cause mortality in NHANES from 1999 to 2018.

	Hazard ratio (95% CI), *P*-value			
	Triglyceride-glucose (TyG) categorical analysis		TyG continuous analysis (per 1-increment)	
Model	Lower TyG index^a^	Higher TyG index^b^	Excluding metabolic factors^e^	Including metabolic factors^f^
Cardiovascular mortality				
Death, no./total no.	712/6,991	141/999	一	一
Model I^c^	Reference	1.56 (1.22, 1.99), 0.0003	1.32 (1.12, 1.56), 0.0008	1.22 (1.03, 1.43), 0.0190
Model II^d^	Reference	1.43 (1.10, 1.85), 0.0067	1.29 (1.08, 1.53), 0.0039	1.18 (1.00, 1.41), 0.0488
All-cause mortality				
Death, no./total no.	1,901/7,680	597/1955	一	一
Model I^e^	Reference	1.20 (1.05 1.38), 0.0086	1.12 (1.03, 1.23), 0.0114	1.11 (1.01, 1.23), 0.0325
Model II^f^	Reference	1.17 (1.02, 1.34), 0.0257	1.11 (1.02, 1.22), 0.0147	1.11 (1.01, 1.22), 0.0339

CI, confidence interval; NHANES, National Health and Nutrition Examination Survey.

a Participants with lower TyG index were <9.5551 and <9.3405 in cardiovascular and all-cause mortality, respectively.

b Participants with lower TyG index were ≥9.5551 and ≥9.3405 in cardiovascular and all-cause mortality, respectively.

c Multivariable-adjusted models were adjusted for age, sex, family income, race, education, marital status.

d Additionally adjusted for body mass index, smoking, drinking, diabetes, hyperlipidaemia, cardiovascular disease, chronic kidney disease, cancer, physical activity, and medications.

e Participants were adjusted for multivariable without blood pressure, blood lipid, and HbA1c.

f Participants were adjusted for multivariable including blood pressure, blood lipid, and HbA1c.

### Joint effects of blood pressure and the triglyceride−glucose index on cardiovascular and all-cause mortality

**Figure 3:**
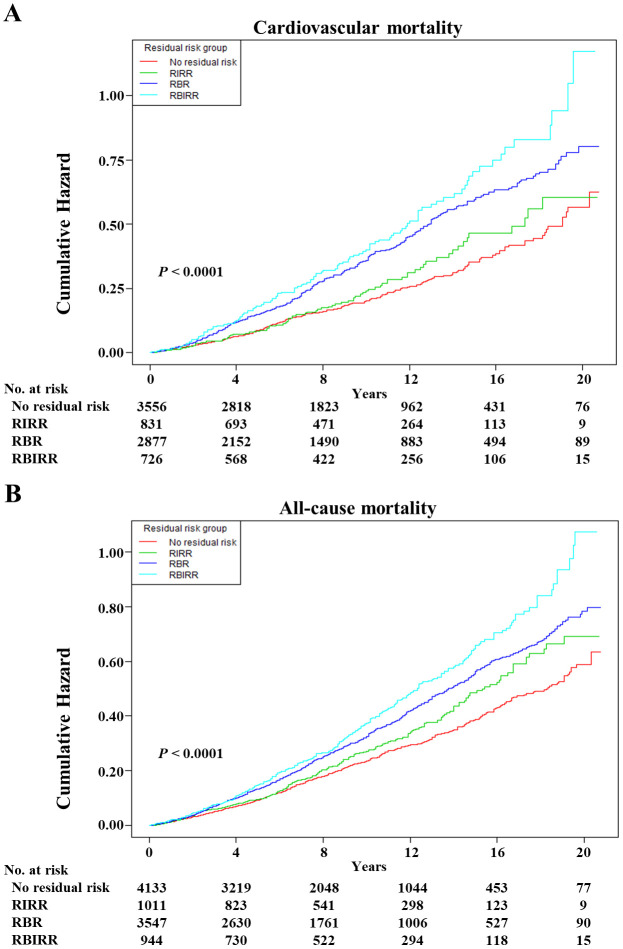
The Kaplan–Meier curves after controlling covariates in **Table 1** for cumulative hazards of the mortality in joint groups. **(A)** Cardiovascular mortality, **(B)** all-cause mortality. RIRR, residual insulin resistance risk, SBP <140 mmHg and DBP <90 mmHg, and with TyG index ≥ estimated threshold; RBR, residual blood pressure risk, SBP ≥140 mmHg or DBP ≥90 mmHg, and with TyG index < estimated threshold; RBIRR, residual blood pressure and insulin resistance risk, SBP ≥140 mmHg or DBP ≥90 mmHg, and with TyG index ≥ estimated threshold.

Kaplan–Meier survival curves were constructed between each joint group, and subsequent mortality risk and log-rank tests with *P* < 0.01 were conducted for the four groups as a whole (**Figure 3**). When either the BP or TyG index remained the same, an increase in the other factor increased the risk of mortality. Clearly, the highest cumulative hazards were observed in the RBIRR group in terms of cardiovascular and all-cause mortality. After adjustment for all potential covariates in the Cox proportional hazards regression models, the associations between residual risk and these mortality endpoints remained (**Table 4**). Compared with those of the no residual risk group, the adjusted HRs of the RIRR, RBR, and RBIRR groups for cardiovascular mortality were 1.44 (1.00, 2.08, *P* = 0.0406), 1.54 (1.23, 1.92, *P* = 0.0001), and 1.64 (1.18, 2.28, *P* = 0.0031), respectively, and those for all-cause mortality were 1.09 (0.91, 1.31), 1.20 (1.04, 1.39, *P* = 0.0098), and 1.39 (1.14, 1.70, *P* = 0.0011), respectively. In general, additive effects of BP and the TyG index were consistently observed for cardiovascular and all-cause mortality.

**Table 4:** Joint analyses of baseline blood pressure and triglyceride-glucose (TyG) index for cardiovascular and all-cause mortality in NHANES from 1999 to 2018.

Model	Hazard ratio (95% CI), *P*-value			
	No residual risk^a^	RIRR^b^	RBR^c^	RBIRR^d^
Cardiovascular mortality				
Death, no./total No.	298/3,859	57/528	414/3,132	84/471
Model I^e^	Reference	1.77 (1.24, 2.52), 0.0018	1.39 (1.13, 1.71), 0.0017	1.93 (1.45, 2.59), <0.0001
Model II^f^	Reference	1.67 (1.16, 2.40), 0.0059	1.53 (1.23, 1.90), 0.0002	1.85 (1.33, 2.58), 0.0003
Model III^g^	Reference	1.44 (1.00, 2.08), 0.0406	1.54 (1.23, 1.92), 0.0001	1.64 (1.18, 2.28), 0.0031
All-cause mortality				
Death, no./total No.	852/4133	260/1011	1049/3547	337/994
Model I^e^	Reference	1.18 (0.99, 1.41), 0.0625	1.15 (1.01, 1.31), 0.0421	1.40 (1.15, 1.71), 0.0008
Model II^f^	Reference	1.12 (0.94, 1.33), 0.2091	1.19 (1.04, 1.36), 0.0129	1.42 (1.16, 1.72), 0.0005
Model III^g^	Reference	1.09 (0.91, 1.31), 0.3404	1.20 (1.04, 1.39), 0.0098	1.39 (1.14, 1.70), 0.0011

CI, confidence interval; NHANES, National Health and Nutrition Examination Survey; RIRR, residual insulin resistance risk; RBR, residual blood pressure risk; RBIRR, residual blood pressure and insulin resistance risk.

a Participants with SBP <140 mmHg and DBP <90 mmHg, and with TyG index < estimated threshold.

b Participants with SBP <140 mmHg and DBP <90 mmHg, and with TyG index ≥ estimated threshold.

c Participants with SBP ≥140 mmHg or DBP ≥90 mmHg, and with TyG index < estimated threshold.

d Participants with SBP ≥140 mmHg or DBP ≥90 mmHg, and with TyG index ≥ estimated threshold.

e Multivariable-adjusted models were adjusted for age, sex, family income, race, education, marital status.

f Additionally adjusted for body mass index, smoking, drinking, diabetes, hyperlipidaemia, cardiovascular disease, chronic kidney disease, cancer, physical activity, and medications.

g Additionally adjusted for metabolic factors including blood lipid and HbA1c.

In sensitivity analyses, the results were generally robust in each joint group when 172 participants who died within 1 year or with a follow-up time of less than 1 year were excluded or when critical BP cut-off values of 130/80 mmHg were used according to the AHA guidelines (with an additional 2,246 hypertensive participants enrolled) (**Supplementary Table S3**). The results remained consistent across multiple subgroup analyses, as summarized in **Supplementary Table S3**. The RBIRR group with the highest HR values for cardiovascular and all-cause mortality was observed among those without obesity and diabetes mellitus regardless of whether or not they were taking antihypertensive drugs.

## Discussion

The main findings of the present large-scale cohort study are as follows. 1) The TyG index predicted elevated cardiovascular and all-cause mortality in hypertension patients with an approximately J-shaped dose−response relationship. The lowest risk thresholds of the TyG index for cardiovascular and all-cause mortality were 9.5551 and 9.3405, respectively. 2) Hypertensive patients who nonetheless have residual risks of both BP and IR had higher mortality and thus, in theory, might benefit from further intervention to control BP and improve IR together. After full adjustment for potential confounders, including age, sex, family income, BMI, race, education, marital status, lifestyle, chronic diseases, medications, and related metabolic factors, the associations of the TyG index and residual risk with mortality remained significant. Additionally, those with residual risks of both BP and IR had higher mortality rates, which was still robust in the sensitivity analyses and was generally consistent across different subgroups without any interaction.

Previous studies have also revealed positive predictability and non-linear relationships between the TyG index and CVD-related adverse outcome and mortality in the general population or various patient groups. For example, Liu et al. demonstrated that the TyG index was U-shaped and associated with incident atrial fibrillation in the general population without known CVD (21). For CVD patients with diabetes or prediabetes, similar U-shaped associations were observed for both CVD and all-cause mortality (22). Nevertheless, it should be mentioned that the lowest risk thresholds of the TyG index for those outcomes in previous studies and our studies remained inconsistent regardless of their similarity. Thus, further research is needed in the future to determine possible unified prediction thresholds. In addition, other studies have explored the positive associations between IR and thyroid hormones and cognitive function in obese and hypertensive populations via the homeostatic model assessment of IR (HOMA-IR) (23, 24). On the one hand, IR may influence hormones, cognitive function, and obesity and thereby mediate hypertension progression. On the other hand, the comparison of the ability of HOMA-IR, a verified marker, and the TyG index to predict mortality among patients with hypertension was significant in our study. However, we could not compare them because personal fasting insulin data were unavailable in the NHANES raw data, and this information is needed to calculate the HOMA-IR with the following formula: fasting glucose × fasting insulin/22.5 (25). Compared with several established IR indices, such as HOMA-IR, HOMA-IS (homeostatic model assessment of insulin sensitivity), and METS-IR (the metabolic score for IR), the TyG index yields superior sensitivity and specificity (4, 26), indicating that it offers distinct advantages over other IR assessment markers.

As such, we believe these data have potential implications for the future treatment of hypertension and the future design of clinical trials. Given the positive correlation coefficients between the TyG index and systolic and diastolic BP, it is currently certain that TyG index is related to IR; in particular, IR can trigger increased BP by activating the RAAS or sympathetic nervous system (7). Clearly, it is necessary and realistic to include diastolic BP in RBR evaluation and joint analyses. As is evident from the present study that the proportion of adults with hypertension who do not take antihypertensive medication and have uncontrolled BP (systolic or diastolic BP ≥140 mmHg or 90 mmHg, respectively) was more than half, indicating that there is urgency and much room for further BP improvement. Interestingly, more than 60% of those with elevated TyG index values took antihypertensive medication, and they were still at increased risk of mortality. This was also supported by the subsequent joint analyses, in which extra RIR caused a 10% to 44% increase in risk compared with equal BP levels; thus, the RBIRR group had the highest risk of cardiovascular and all-cause mortality. These findings suggest that simply taking medication to control BP or inhibit RAAS activation might not completely counteract the risk of death caused by increased IR. In addition to the above influences, IR can induce oxidative stress, exacerbate inflammatory responses, and impair endothelial function, leading to hyperglycaemia or dyslipidaemia (27, 28). Moreover, IR in individuals with prediabetes plays substantial roles in the development of cognitive and physical impairments, which increase the risk of frailty and eventually limit the management of hypertension (29). All of these factors could contribute to the progression and poor outcomes of hypertension. In addition, hypertension patients who maintained a normal BP (<140/90 mmHg or even <130/80 mmHg) but had higher levels of IR had a 1.44-times higher risk of cardiovascular death than were those with normal BP and IR levels; indeed, these individuals need more active intervention because they always feel that they are at a low risk. Overall, we propose the emerging hypothesis that combination therapies that lower BP and improve IR may be beneficial for reducing mortality in the hypertensive population.

As mentioned in the background section, a previous study failed to find any significant associations between mortality and joint analysis groups on the basis of the 140/90 mmHg hypertension cut-off. However, in the present research, these associations were positive regardless of whether the hypertension cut-off was 140/90 mmHg or 130/80 mmHg. Similarly, these additive effects in the joint analyses were not influenced by hypertensive patients whether or not they were on antihypertensive therapy. Notably, the TyG index was initially calculated from triglyceride and glycaemic parameters in a healthy population (30). Therefore, the TyG index is likely affected by metabolic factors, including obesity and diabetes. In fact, those with obesity or without diabetes were not observed to have similar associations according to the subgroup analysis results. Overall, the application of the TyG index might be suitable for the hypertensive population because it does not interfere with the diagnostic cut-off or the use of antihypertensive medication.

### Strengths and limitations

Our research has several strengths. To our knowledge, this is the largest sample size real-world study on the associations of the TyG index and its combination with BP with cardiovascular and all-cause mortality in hypertension patients. The TyG index in this study is based on measurements that are easily available early in routine laboratory analyses in most hospitals, which is more convenient for monitoring personal IR levels. Additionally, a nationally representative sample of American adults with hypertension was analysed in the present study, which facilitates the generalization of our findings. However, the present study also has some inevitable limitations. First, a single baseline record of the TyG index and BP was available in NHANES, and their fluctuations were not considered in our study, which prevented further investigation of the time−course associations between their changes and mortality. Second, residual or unknown confounders might exist and interfere with our results. Third, we could not deduce causal conclusions owing to the nature of the observational study design. Thus, the results should be interpreted with caution in clinical practice.

## Conclusion

Our data indicate that the TyG index is a convenient and valuable biomarker for predicting the risk of cardiovascular and all-cause death in patients treated with hypertension. The dual residual risk of BP and IR was associated with the highest increased risk of cardiovascular and all-cause mortality; thus, we propose that future combination therapies targeting both the BP and IR pathways could improve patient care, disease progression, and residual risk. Nevertheless, well-designed randomized clinical trials are needed to further assess our findings.

## Data Availability

The original contributions presented in the study are included in the article/**Supplementary Material**. Further inquiries can be directed to the corresponding authors.
