# Supplementary material for: The associations of the triglyceride-glucose index and its combination with blood pressure on cardiovascular and all-cause mortality in hypertension: a national study
Source: Front Endocrinol (Lausanne). 2024 Oct 14;15:1469055. doi: 10.3389/fendo.2024.1469055 (PMC11513261; doi:10.3389/fendo.2024.1469055)
Supplement: Supplementary file 1 [file DataSheet1.docx]

Supplementary Table 1. Effect of polyhalite based fertilizer application on growth and yield attributes of groundnut in two locations during 2021-22 and 2022-23.

| Treatment | Germination (%) | | 100 seed weight (g) | | Pods plant^-1^ | | Shelling % | |  |
| --- | --- | --- | --- | --- | --- | --- | --- | --- | --- |
|  | 2021-22 | 2022-23 | 2021-22 | 2022-23 | 2021-22 | 2022-23 | 2021-22 | 2022-23 |  |
| *Location* | | | | | | | | | |
| Hyderabad | 80.2a | 80.6a | 38.2b | 32.6b | 40.8a | 33.6a | 68.0b | 64.7a |  |
| Ananthapur | 78.7a | 79.4b | 41.5a | 42.5a | 11.0b | 11.1b | 70.0a | 65.6a |  |
| *Fertilizer sources* | | | | | | | | | |
| Rec. NPK + gypsum @ 500 kg ha^-1^ | 80.5a | 80.5a | 39.8ab | 39.6a | 26.7 | 23.7ab | 69.5ab | 66.9abc |  |
| Rec. NP only | 77.9a | 78.9a | 39.4bc | 35.5d | 24.2cd | 21.5e | 69.1ab | 62.9fg |  |
| Rec. NPK | 80.1a | 81.1a | 39.2bc | 37.8bc | 26.0abc | 23.2abc | 67.4bc | 65.2cde |  |
| Rec. NP + 50% K through MOP | 79.8a | 79.9a | 39.7b | 36.5cd | 25.6abc | 21.8cde | 69.5ab | 63.5ef |  |
| Rec. NP + 100% K through Poly4 | 80.2a | 79.3a | 42.1a | 39.2ab | 27.3a | 24.1a | 71.4a | 68.0ab |  |
| Rec. NP + 100% K through Poly4 + gypsum @ 310 | 80.1a | 80.2a | 41.2ab | 40.2a | 27.1ab | 23.8a | 71.7a | 68.5a |  |
| Rec. NP + 50% K through Poly4 | 79.3a | 81.1a | 40.4ab | 37.6c | 26.3abc | 22.3bcde | 70.5a | 65.2cde |  |
| Rec. NP + 50% K through Poly4 + gypsum @ 310 | 79.8a | 80.0a | 39.9ab | 39.3ab | 27.1ab | 23.0abcd | 69.2ab | 66.0bcd |  |
| Rec. NP + gypsum @ 500 kg ha^-1^ | 79.5a | 80.6a | 39.6b | 37.2c | 25.3bc | 21.7de | 67.2bc | 64.6def |  |
| Control | 77.6a | 78.1a | 37.2c | 32.2e | 23.3d | 18.5f | 64.7c | 60.9g |  |

Supplementary Table 2. Effect of polyhalite based fertilizer application on pod, haulm and kernel yields of groundnut in two locations during 2021-22 and 2022-23.

| Treatment | Pod yield  (Mg ha^-1^) | | Haulm yield (Mg ha^-1^) | | Kernel yield  (Mg ha^-1^) | | Harvest index (%) | | Oil yield  (Mg ha^-1^) | |  |
| --- | --- | --- | --- | --- | --- | --- | --- | --- | --- | --- | --- |
|  | 2021-22 | 2022-23 | 2021-22 | 2022-23 | 2021-22 | 2022-23 | 2021-22 | 2022-23 | 2021-22 | 2022-23 |  |
| *Location* | | | | | | | | | | | |
| Hyderabad | 2.19a | 1.38a | 2.15b | 1.85b | 1.49a | 0.90a | 50.3a | 42.6a | 0.78a | 0.44a |  |
| Ananthapur | 1.46b | 1.51a | 3.33a | 3.71a | 1.02b | 1.00a | 44.0b | 40.5b | 0.49b | 0.49a |  |
| *Fertilizer sources* | | | | | | | | | | | |
| Rec. NPK + gypsum @ 500 | 1.86bcd | 1.66a | 2.68cd | 2.96a | 1.29bc | 1.11ab | 47.9bc | 44.5ab | 0.72ab | 0.55a |  |
| Rec. NP only | 1.69e | 1.27ad | 2.75abc | 2.74a | 1.16d | 0.80f | 45.2cd | 38.1d | 0.53e | 0.39e |  |
| Rec. NPK | 1.84bcd | 1.51abc | 2.70cd | 2.88a | 1.24cd | 0.99cd | 47.6bc | 41.9bc | 0.64cd | 0.48cd |  |
| Rec. NP + 50% K through MOP | 1.81cd | 1.37cd | 2.92a | 2.76a | 1.25cd | 0.87ef | 45.0cd | 39.6cd | 0.59de | 0.43de |  |
| Rec. NP + 100% K through Poly4 | 2.00a | 1.65a | 2.73bcd | 2.82a | 1.42a | 1.13a | 50.3ab | 46.1a | 0.75a | 0.56a |  |
| Rec. NP + 100% K through Poly4 + gypsum @ 310 | 1.94ab | 1.58ab | 2.55d | 2.93a | 1.38ab | 1.09abc | 51.9a | 42.8abc | 0.73ab | 0.55ab |  |
| Rec. NP + 50% K through Poly4 | 1.90abc | 1.52abc | 2.77abc | 2.89a | 1.33abc | 0.99bcd | 48.1bc | 42.2bc | 0.66bc | 0.49bc |  |
| Rec. NP + 50% K through Poly4 + gypsum @ 310 | 1.88abc | 1.49bc | 2.70cd | 2.77a | 1.30bc | 0.99cd | 48.1bc | 42.8abc | 0.67bc | 0.49c |  |
| Rec. NP + gypsum @ 500 | 1.76de | 1.48bc | 2.90ab | 2.72a | 1.18d | 0.95de | 44.1d | 43.8ab | 0.63cd | 0.47cd |  |
| Control | 1.55f | 0.94e | 2.69cd | 2.34b | 1.01e | 0.57g | 43.7d | 34.0e | 0.43f | 0.27f |  |

Supplementary Table 3. Effect of polyhalite based fertilizer application on oil % and quality parameters of groundnut in two locations during 2021-22 and 2022-23.

| Treatment | Oil content (%) | | Oleic acid (%) | | Linoleic acid (%) | | Palmitic acid (%) | | Stearic acid (%) | |
| --- | --- | --- | --- | --- | --- | --- | --- | --- | --- | --- |
|  | 2021-22 | 2022-23 | 2021-22 | 2022-23 | 2021-22 | 2022-23 | 2021-22 | 2022-23 | 2021-22 | 2022-23 |
| *Location* | | | | | | | | | | |
| Hyderabad | 52.2a | 49.1a | 48.53a | 46.9a | 44.1a | 37.2b | 10.90a | 11.07a | 3.13a | 2.45a |
| Ananthapur | 48.7b | 48.4a | 41.5b | 40.4b | 34.6b | 44.1a | 9.47b | 11.09a | 2.51b | 2.50a |
| *Fertilizer sources* | | | | | | | | | | |
| Rec. NPK + gypsum @ 500 | 51.2abc | 48.8bcd | 46.1b | 42.9cd | 39.1abc | 41.6ab | 10.38ab | 11.04ab | 2.96ab | 2.41bcd |
| Rec. NP only | 49.4ef | 48.3cd | 41.1d | 41.7cd | 41.1a | 43.1a | 10.03bc | 11.30a | 3.03a | 2.47bc |
| Rec. NPK | 49.9de | 49.0bc | 45.1bc | 42.6cd | 41.9a | 41.1ab | 10.20ab | 11.13ab | 2.67ab | 2.53ab |
| Rec. NP + 50% K through MOP | 50.6cd | 48.7bcd | 42.2cd | 47.9a | 41.6a | 36.7cd | 10.21ab | 10.46bc | 2.62b | 2.56ab |
| Rec. NP + 100% K through Poly4 | 51.4ab | 49.1b | 47.8ab | 43.1cd | 39.8a | 41.1ab | 10.59ab | 11.56a | 2.82ab | 2.57ab |
| Rec. NP + 100% K through Poly4 + gypsum @ 310 | 51.6a | 49.0bc | 50.1a | 47.3ab | 39.8ab | 35.9d | 10.71a | 11.50a | 2.89ab | 2.87a |
| Rec. NP + 50% K through Poly4 | 50.8bc | 48.2d | 45.5b | 42.8cd | 35.8c | 40.9ab | 10.38ab | 11.49a | 3.00ab | 2.56ab |
| Rec. NP + 50% K through Poly4 + gypsum @ 310 | 50.5cd | 49.9a | 45.5b | 44.4bc | 39.6ab | 39.8bc | 10.66ab | 11.09ab | 2.77ab | 2.59ab |
| Rec. NP + gypsum @ 500 kg ha^-1^ | 50.6cd | 48.5bcd | 45.4b | 40.8d | 38.5abc | 43.9a | 9.52cd | 11.23a | 2.62b | 2.10d |
| Control | 49.1f | 48.3cd | 41.4d | 43.4cd | 36.4bc | 42.5ab | 9.14d | 9.97c | 2.85ab | 2.16cd |

Supplementary Table 4. Effect of polyhalite based fertilizer application on soil organic carbon (SOC) and soil available nutrient status after harvesting of groundnut in two locations during 2021-22 and 2022-23.

| Treatment | SOC (%) | | Available N (mg kg^-1^) | | Available P (mg kg^-1^) | | Available K (mg kg^-1^) | |  |
| --- | --- | --- | --- | --- | --- | --- | --- | --- | --- |
|  | 2021-22 | 2022-23 | 2021-22 | 2022-23 | 2021-22 | 2022-23 | 2021-22 | 2022-23 |  |
| *Location* | | | | | | | | | |
| Hyderabad | 0.46 | 0.47a | 130.4a | 130.4a | 42.9a | 40.0a | 123.6b | 123.0a |  |
| Ananthapur | 0.34b | 0.35b | 116.2b | 119.7b | 13.9b | 13.9b | 164.2a | 162.9a |  |
| *Fertilizer sources* | | | | | | | | | |
| Rec. NPK + gypsum @ 500 kg ha^-1^ | 0.41a | 0.43a | 126.7a | 128.7a | 30.4a | 28.9a | 152.6a | 151.9a |  |
| Rec. NP only | 0.39bc | 0.39cd | 118.5cd | 120.3cd | 27.1c | 25.6c | 134.5e | 132.6e |  |
| Rec. NPK | 0.40abc | 0.40bcd | 125.0 | 126.8ab | 29.5ab | 28.1ab | 147.4abc | 146.9abc |  |
| Rec. NP + 50% K through MOP | 0.40abc | 0.41abc | 119.6bc | 121.6bc | 28.1bc | 26.6bc | 143.9cd | 142.7cd |  |
| Rec. NP + 100% K through Poly4 | 0.41ab | 0.41abc | 125.7 | 127.4a | 29.2ab | 27.7ab | 151.6a | 151.1a |  |
| Rec. NP + 100% K through Poly4 + gypsum @ 310 kg ha^-1^ | 0.42a | 0.43a | 127.0a | 128.7a | 29.4ab | 28.0ab | 150.7ab | 150.2ab |  |
| Rec. NP + 50% K through Poly4 | 0.41ab | 0.41abc | 125.6a | 127.5a | 27.6bc | 26.2bc | 148.8abc | 148.0abc |  |
| Rec. NP + 50% K through Poly4 + gypsum @ 310 kg ha^-1^ | 0.41ab | 0.42ab | 123.9ab | 125.7ab | 28.7abc | 27.3abc | 145.abc | 144.5bc |  |
| Rec. NP + gypsum @ 500 kg ha^-1^ | 0.40abc | 0.41abc | 126.5a | 128.2a | 29.3ab | 27.9ab | 137.7de | 136.4de |  |
| Control | 0.38c | 0.38d | 114.3d | 115.6d | 24.9d | 23.3d | 126.3f | 125.3f |  |

Supplementary Table 5. Effect of polyhalite based fertilizer application on soil micronutrient status after harvesting of groundnut in two locations during 2021-22 and 2022-23.

| Treatment | Cu (mg kg^-1^) | | Fe (mg kg^-1^) | | Zn (mg kg^-1^) | | Mn (mg kg^-1^) | |
| --- | --- | --- | --- | --- | --- | --- | --- | --- |
|  | 2021-22 | 2022-23 | 2021-22 | 2022-23 | 2021-22 | 2022-23 | 2021-22 | 2022-23 |
| *Location* | | | | | | | | |
| Hyderabad | 1.61a | 1.62a | 8.65b | 8.61b | 0.46b | 0.46b | 21.12b | 21.1b |
| Ananthapur | 0.78b | 0.99b | 21.6a | 22.9a | 1.32a | 1.43a | 39.4a | 41.7a |
| *Fertilizer source* | | | | | | | | |
| Rec. NPK + gypsum @ 500 kg ha^-1^ | 1.24a | 1.34a | 15.8ab | 16.4ab | 1.02a | 1.08a | 28.96ab | 30.1ab |
| Rec. NP only | 1.17a | 1.31a | 14.3cd | 15.0cd | 0.85bcd | 0.91bc | 31.86a | 33.0a |
| Rec. NPK | 1.13a | 1.24a | 14.8bcd | 15.4bcd | 0.92abcd | 0.97ab | 30.34ab | 31.5ab |
| Rec. NP + 50% K through MOP | 1.12a | 1.23a | 15.0abcd | 15.7abcd | 0.79cd | 0.85bc | 30.11ab | 31.2ab |
| Rec. NP + 100% K through Poly4 | 1.33a | 1.41a | 15.56ab | 16.2ab | 0.94ab | 0.99ab | 31.71a | 32.9a |
| Rec. NP + 100% K through Poly4 + gypsum @ 310 | 1.18a | 1.30a | 15.70ab | 16.4ab | 1.02a | 1.08a | 28.99ab | 30.1ab |
| Rec. NP + 50% K through Poly4 | 1.14a | 1.28a | 16.01a | 16.6a | 0.93abc | 0.98ab | 29.13ab | 30.2ab |
| Rec. NP + 50% K through Poly4 + gypsum @ 310 | 1.17a | 1.30a | 15.03abcd | 15.7abcd | 0.87bcd | 0.93bc | 31.87a | 33.0a |
| Rec. NP + gypsum @ 500 kg ha^-1^ | 1.29a | 1.37a | 15.23abc | 15.9abc | 0.82bcd | 0.88bc | 32.41a | 33.6a |
| Control | 1.21a | 1.29a | 14.08d | 14.6d | 0.78d | 0.82c | 27.40b | 28.5b |

Supplementary Table 6. Effect of poly4 based fertilizer application on profitability of groundnut in two locations during 2021-22 and 2022-23.

| Treatment | Cost of cultivation (US$ ha^-1^) | | Gross returns (US$ ha^-1^) | | Net returns (US$ ha^-1^) | | B:C | |  |
| --- | --- | --- | --- | --- | --- | --- | --- | --- | --- |
|  | 2021-22 | 2022-23 | 2021-22 | 2022-23 | 2021-22 | 2022-23 | 2021-22 | 2022-23 |  |
| *Location* | | | | | | | | | |
| Hyderabad | 497a | 497a | 1558a | 1000a | 1061a | 503b | 2.13a | 1.01a |  |
| Ananthapur | 497a | 506a | 1101b | 1148a | 605b | 642a | 1.22b | 1.26a |  |
| *Fertilizer source* | | | | | | | | | |
| Rec. NPK + gypsum @ 500 | 503a | 507a | 1354bc | 1222a | 851abc | 714a | 1.69abcd | 1.41a |  |
| Rec. NP only | 466a | 471a | 1239d | 954e | 773c | 482d | 1.66bcde | 1.02d |  |
| Rec. NPK | 474a | 479a | 1338bc | 1122abcd | 863ab | 643abc | 1.82a | 1.34ab |  |
| Rec. NP + 50% K through MOP | 472a | 476a | 1329bc | 1018de | 857ab | 542cd | 1.82ab | 1.14bcd |  |
| Rec. NP + 100% K through Poly4 | 532a | 536a | 1448a | 1216ab | 916a | 679ab | 1.73abc | 1.27abc |  |
| Rec. NP + 100% K through Poly4 + gypsum @ 310 | 551a | 555a | 1405ab | 1172abc | 854abc | 616abc | 1.55de | 1.11cd |  |
| Rec. NP + 50% K through Poly4 | 499a | 504a | 1382ab | 1127abcd | 883a | 623abc | 1.77abc | 1.24bcd |  |
| Rec. NP + 50% K through Poly4 + gypsum @ 310 | 518a | 523a | 1365bc | 1105bcd | 847abc | 582bcd | 1.63cde | 1.11cd |  |
| Rec. NP + gypsum @ 500 | 495a | 499a | 1290cd | 1093cd | 795bc | 594bcd | 1.61cde | 1.19bcd |  |
| Control | 457a | 462a | 1146e | 714f | 688d | 252e | 1.51e | 0.56e |  |

Supplementary figure 1. Weekly weather data of ICAR-CRIDA, Hyderabad during (a) 2021 and (b) 2022 crop growth period.

Supplementary figure 2. Weekly weather data of ICAR-DGR, Ananthapur during (a) 2021 and (b) 2022 crop growth period.


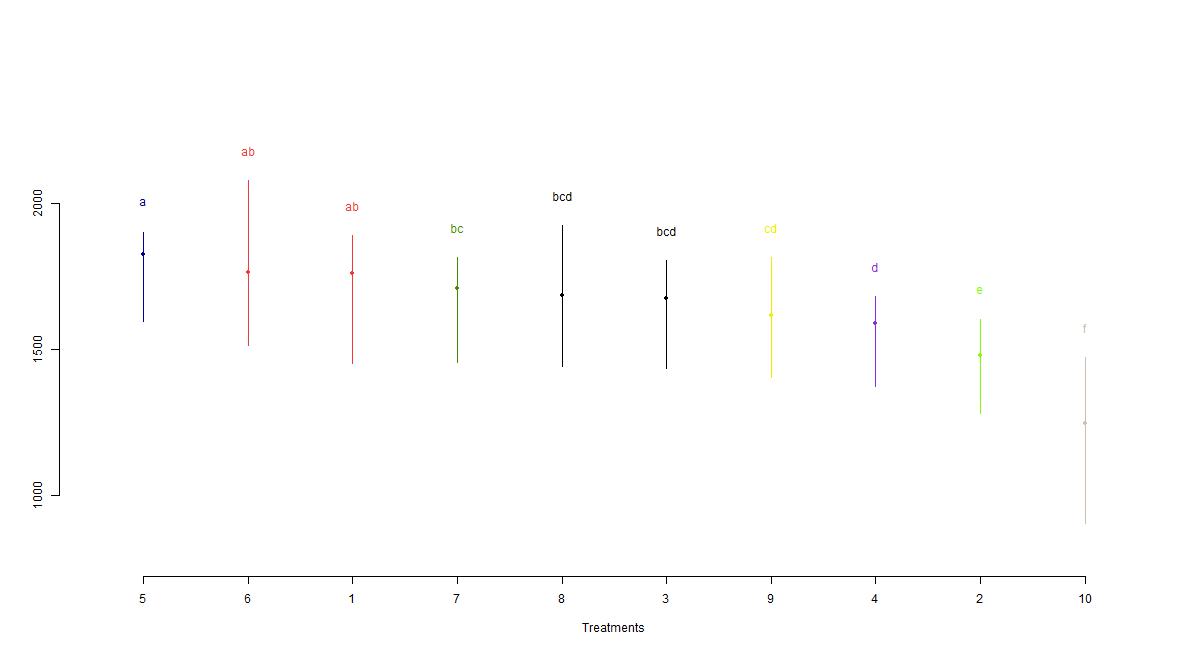


Supplementary figure 3: Variation in pooled pod yield of groundnut under various fertilizer sources


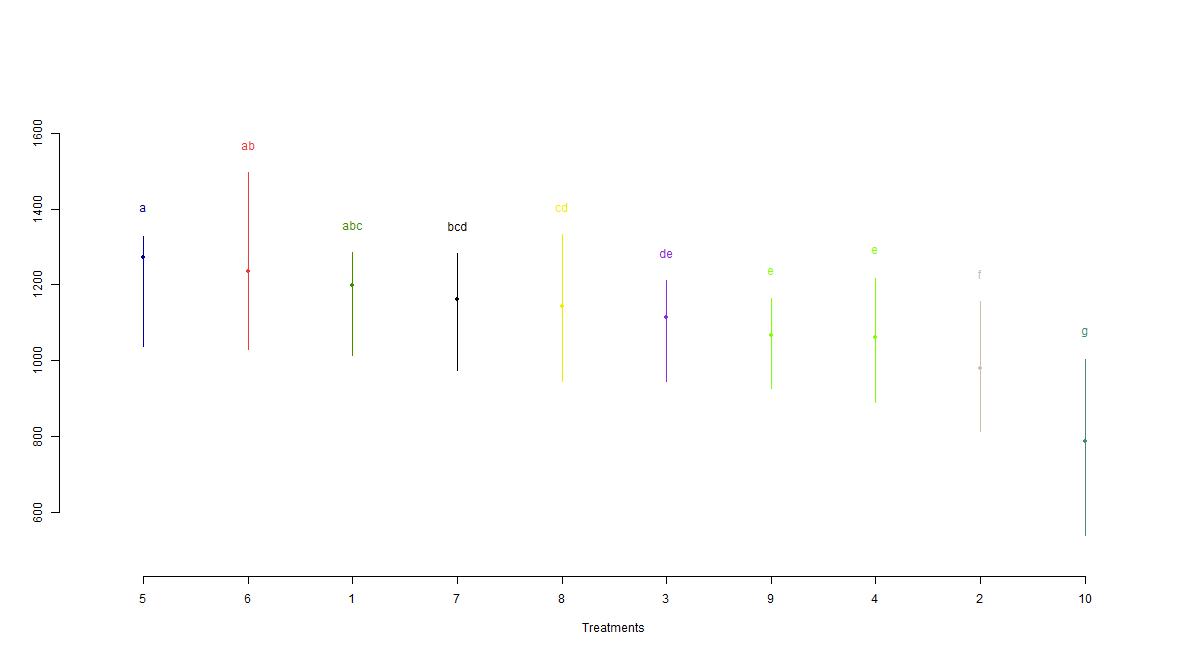


Supplementary figure 4: Variation in pooled kernel yield of groundnut under various fertilizer sources
